# Study protocol: Developing telephone follow-up scale for patients with disorders of consciousness

**DOI:** 10.3389/fpubh.2023.1071008

**Published:** 2023-03-30

**Authors:** Fangfang Shou, Jing Wang, Steven Laureys, Lijuan Cheng, Wangshan Huang, Haibo Di

**Affiliations:** ^1^International Unresponsive Wakefulness Syndrome and Consciousness Science Institute, Hangzhou Normal University, Hangzhou, China; ^2^Faculty of Nursing, Hangzhou Normal University, Hangzhou, China; ^3^Coma Science Group, GIGA-Consciousness, University of Liege, Liege, Belgium; ^4^Centre du Cerveau, University Hospital of Liege, Liege, Belgium

**Keywords:** disorders of consciousness, prognosis, follow-up tool, telephone follow-up, diagnosis

## Abstract

**Background:**

The significant lack of rehabilitation prognostic data is the main reason that affects the treatment decision-making and ethical issues of patients with disorders of consciousness (DoC). Currently, the clinic's consciousness assessment tools cannot satisfy DoC patients' follow-up needs.

**Objective:**

The purpose of this study is to construct a sensitive, professional, and simple telephone follow-up scale for DoC patients to follow up on the prognosis, especially the recovery of consciousness, of prolonged DoC patients transferred to community hospitals or at home.

**Methods:**

This study is to adopt expert consultation to construct and to verify the validity and feasibility of the scale on-site.

**Conclusion:**

At present, there is a strong demand for portable, accurate, and easily operated scales. It is helpful to improve the rehabilitation data of prolonged DoC patients and provide more basis for their treatment and rehabilitation.

## Introduction

With the progress of medical technology and the development of artificial feeding, more and more patients with Disorders of Consciousness (DoC) have survived. Among the broad classification of DoC (1), three main conscious levels have been clinically identified according to behavioral criteria: Coma, Unresponsive Wakefulness Syndrome (UWS) or Vegetative State (VS), and Minimally Conscious State (MCS) (2). Coma Recovery Scale-Revised (CRS-R) is considered as the gold standard for consciousness assessment (3, 4) and the primary method for consciousness follow-up. CRS-R includes auditory, visual, motor, oral, communication and arousal (5, 6). Such patients can be divided into coma, UWS/VS, MCS-, MCS+, and Emergence from the Minimally Conscious State (EMCS) according to their behaviors (7). However, CRS-R operation is conducted face-to-face, which is time-consuming. Furthermore, due to the fluctuation of awakening and the effect of drugs, it is difficult to accurately identify the patient of consciousness in a one-time behavioral assessment. Studies have shown that repeated CRS-R assessments can reduce misdiagnosis (8). However, for community hospitals and home-based patients, implementing behavioral assessment is difficult due to transportation and time cost, especially for low-income families or assessors.

In the past decades, telephone follow-up was increasingly utilized in various fields. In the medical area, it has been variously used for patient compliance and continuity of care after patient discharged (9–14). Telephone follow-up can comprehensively assess patients' state of consciousness and provide remote health education for patients and their family members. Therefore, a highly targeted and feasible telephone follow-up scale is urgently needed.

Telephone service is an innovative expansion of the traditional “face-to-face” medical service, which is timelier and more flexible, more personalized, and more convenient to operate (15). Patients can communicate with healthcare workers anytime and anywhere through cell phones or other mobile platforms. Medical staffs can use the telephone to follow up, inform patients and exchange pictures and images sent by patients for preliminary diagnosis and medical guidance, which can overcome the time and space limitations of medical services (16). Telephone follow-up is to guide and supervise patients' condition and recovery status and psychological status *via* telephone. The purpose is to enable patients to continue to receive health education and medical services even after discharged. This service can effectively extend the care in-hospital to out-of-hospital. In addition, for DoC patients, the telephone program allows not only voice communication but also video assessment, which can keep abreast of the prognosis status and disease progression of DoC patients and improve patients' self-management ability, thus reducing the hospitalization rate and mortality rate of related diseases.

This study aims to construct a telephone follow-up scale more suitable for DoC patients. The scale is constructed using the currently verified consciousness indicators [vision pursuit (2), auditory localization (17), reproducible response to command, automatic motor response (18), localization to harmful stimuli (19) and olfactory stimulus response (20, 21)] observed by the patients' family members. The telephone consultant will make a consciousness diagnosis according to the information provided by the patient's family members.

## Materials and methods

### Study design

**Figure 1:**
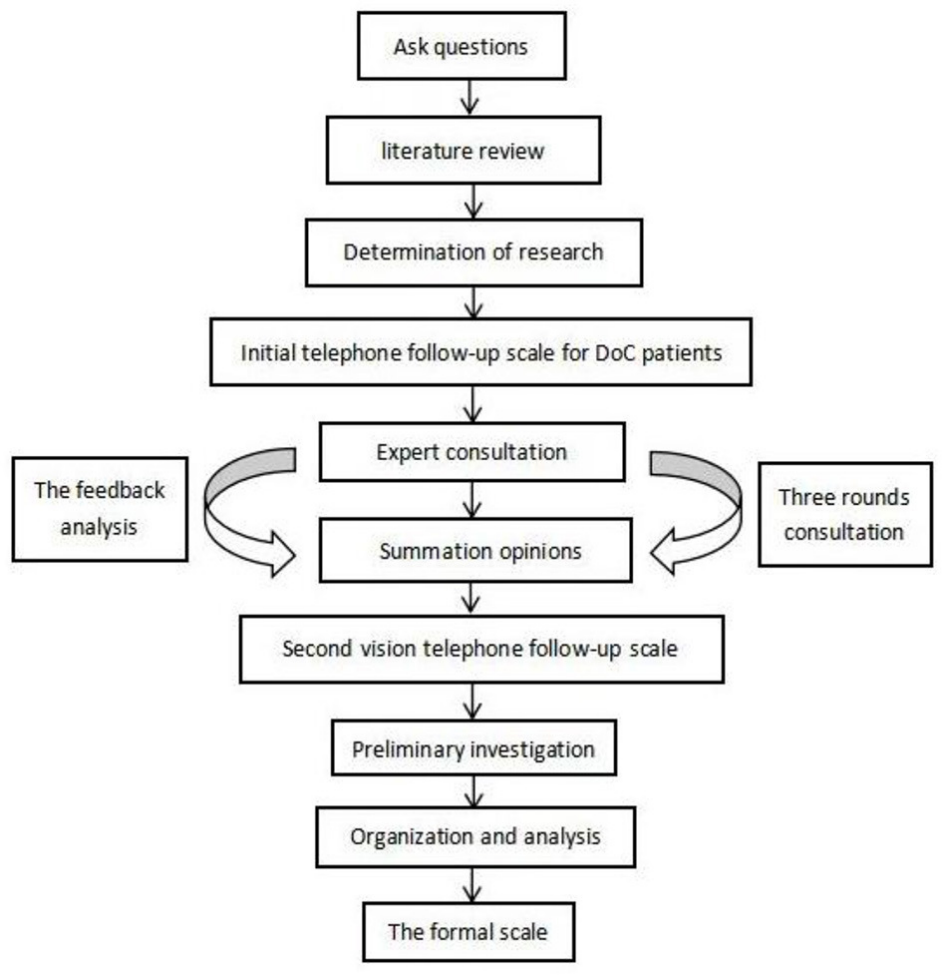
Flow chart of scale development.

**Figure 2:**
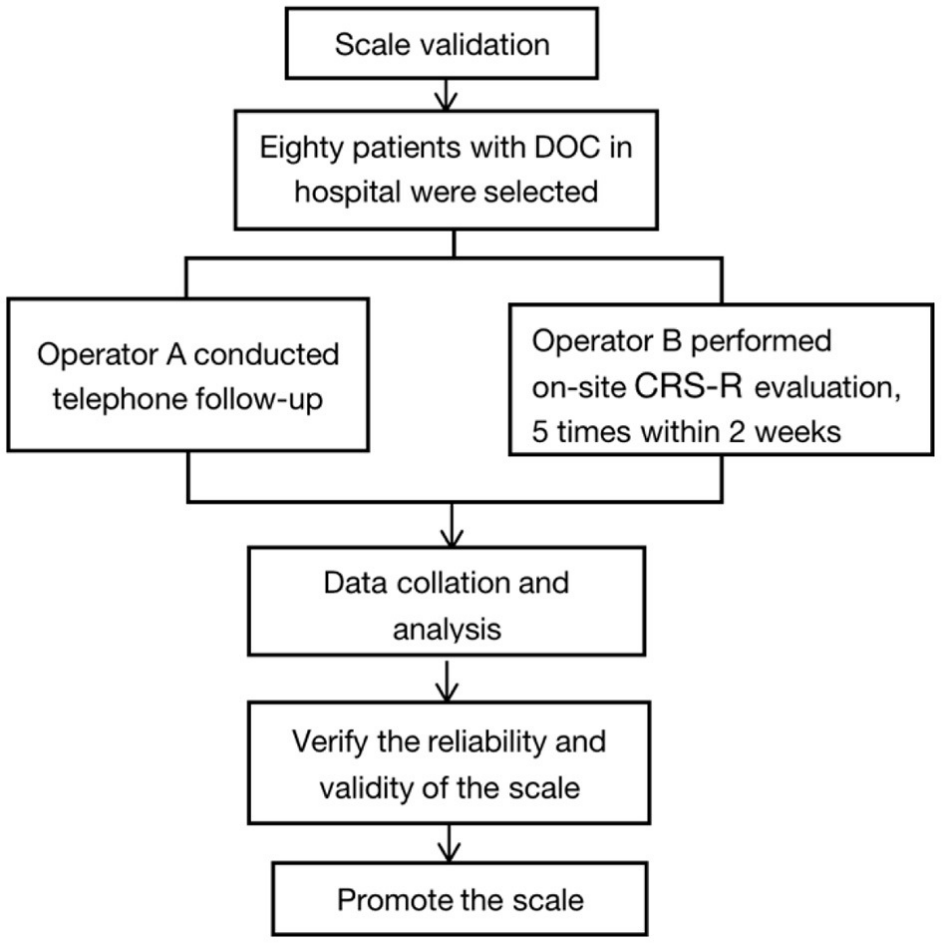
Flow chart of scale validation.

This study is divided into two stages (see Figures 1, 2).

Stage 1: To develop the telephone follow-up scale for consciousness recovery.
Stage 2: To verify the accuracy and validity of the telephone follow-up scale for patients with DoC.

### Establishment of DoC telephone follow-up scale construction team

The team members are divided into two groups. One is the study group, consisting of professionally trained researchers and clinicians. The other is the expert group, consisting of researchers and clinicians, doctors, and professional statisticians with over 10 years of experience in DoC research.

### Stage 1

DoC telephone follow-up scale will be used to investigate the prognosis and recovery in eight points: awakening, vision, hearing, communication, movement, touch or pain sensation, smell or taste, emotional response, and functional recovery.

### Draft construction

The draft of the telephone follow-up scale was developed based on published literature, DoC field-related guidelines, and clinical scales.

### Expert consultation

#### First round: Expert consultation

The first round was done face-to-face and email communication with the expert group. Firstly, we will briefly introduce the purpose and significance of this study and invite experts to answer the questionnaires, which are divided into two parts: The first part is the preliminary framework of the telephone follow-up program for DoC patients' consciousness recovery (see Appendix 1). The second part is the questionnaire of experts (see Appendix 2). In the questionnaire, experts will score the importance, accuracy, and operability of all indicators of scale items, write corresponding scores in the blanks, and put forward suggestions for modification. And the opinion collation, induction, and statistics.

#### Second round: Expert consultation

The language will be revised according to the items determined in the first round. The revised scale and opinions will be fed back to the expert group for the second round of expert consultation. The main content of the second round includes re-evaluation of the selected items and the understanding from patient's family members on such items. The expert opinions of this round will be sorted out, summarized, counted, and fed back again.

#### Third round: Expert consultation

The scale will be revised according to the second round of expert feedback. The opinions were synthesized and fed back to the expert group for the third round of expert consultation. After feedback, a study group discussion was conducted to form a second vision telephone follow-up scale.

### The preliminary investigation

The pre-survey sample size will be 10–20% of the total sample size. Ten patients will be selected for a pre-test of the telephone follow-up scale at the hospital. The main investigation content includes whether the family members can understand the entry. The result will be used to revise the final version of the telephone follow-up scale. According to the telephone follow-up scale's pre-survey results, the final version of the telephone follow-up scale will be revised.

### Stage 2

#### Patient selection

##### Sample size

The formula is “N = [number of variables × (5–10) ^*^ (1–15%)]”. According to the Kendall sample size calculation method, the sample size should be 5–10 times the number of independent variables, and a 15% missing rate should be set.

#### Patients

Inclusion criteria: (1) Patients with brain injury for over 28 days. (2) Age between 18 years old and 65 years old. (3) No sedatives administered within 15 days. (4) No fever, infection, or other symptoms occurred within 15 days. (5) The agreement from patients' families on participation in the study with signed informed consent.

Exclusion criteria: (1) The patient has documented dysfunction resulting from developmental psychosis or neurological disease. (2) Patients with untreated epilepsy. (3) Untreated cerebral edema. (4) The patient's vital signs are unstable.

#### Patient's primary caregiver

Inclusion criteria: (1) Undertaking primary care of the patients. (2) Age over 18 years. (3) Caregiving time over 3 hours per day. (4) Agree to participate in the study and sign an informed consent form.

Exclusion criteria: (1) Caregivers with cognitive or psychiatric disorders who cannot communicate properly. (2) Those who refused to participate in this study or withdrew during the experimental study.

#### Scale verification

The primary caregivers of DoC patients are first trained on the scale. The main content of the training includes observation indicators and operation specifications. Family members of discharged patients will receive operation videos to guide their observations. Once the family observed the target behavior, a video was taken and sent to researcher A. After 2 weeks, Investigator A followed up with the patients by telephone or video and diagnosed their consciousness. Investigator B assessed patients face-to-face using CRS-R, the behavioral gold standard.

### Diagnostic criteria

The project evaluation criteria mainly refer to the CRS-R evaluation criteria for MCS and EMCS. In CRS-R evaluation, functional communication and object use can be diagnosed as EMCS. Reproducible movement to command and object recognition is MCS+. Visual pursuit, automatic motor response and pain localization as MCS-. These behaviors can be observed frequently (more than three times), occasionally or none. Only frequently observed behaviors are rated. For occasionally observed behaviors, the patients will be further observed by their families. The follow up is going to be carried out in every 2 weeks until the patient recovers from EMCS or dies.

### Statistical analysis

The telephone follow-up scale was designed to effectively assess the recovery of DoC patients without the presence of a professional for the face-to-face assessment. Therefore, the diagnosis of consciousness is not based on the total score of the scale but on whether patients show repeatable conscious behavior.

#### Feasibility assessment

The actual and average use time of telephone follow-up and the satisfaction of patients' family members will be collected.

#### Internal consistency

SPSS 27.0 statistical software will be used for statistical analysis: Calculate Cronbach's α coefficient and half-fold reliability of each dimension and total amount table. Cronbach's α > 0.65 is normal. Cronbach's α > 0.85 is good.

#### Retest reliability

20 of the 80 patients will be selected for evaluation 2 weeks later, and the retest correlation coefficient r will be calculated, ranging from 0 to 1. The closer r is to 1, the higher the retest reliability is.

#### Content validity

Five experts in the field of DoC will be invited to evaluate the content validity I-CVI for each item. When the value of I-CVI reached more than 0.78, the research tool is considered to have good content validity.

#### Convergence validity

Cohen Weighted Kappa (22) will be used to assess the correlation between CRS-R and the telephone follow-up scale.

#### Structural validity

Exploratory factor analysis is performed using principal component analysis and variance maximization orthogonal rotation method. If the cumulative explanation of common factors in the scale is > 50% variation, the number of common factors is consistent with the theoretical hypothesis. The loading value of each item on the corresponding common factors is high (> 0.4), and the loading value on other common factors is low, which can be considered that the scale has good structural validity.

## Discussion

Electrophysiological and imaging methods cannot be used as long-term follow-up tools for DoC patients due to their high cost, complexity, and contraindications. Therefore, behavioral scales remain the most basic assessment tool and have irreplaceable clinical value (23). The CRS-R is currently the most widely used clinical assessment (24) and follow-up tool (25). However, the disadvantage is that it is time-consuming and requires face-to-face evaluation by medical personnel. In addition, evaluating CRS-R in rehabilitation hospitals, community hospitals, and home-based patients is more complicated and time-consuming. In addition, there are currently Glasgow coma scale (GCS) (26), Full outline unresponsive (FOUR) (27), Wessex head injury matrix (WHIM) (28), Sensory modality assessment and rehabilitation technique (SMART) (29), Western neuro sensory stimulation profile (WNSSP) (30) and Disorder of consciousness scale (DOCS) (31) in the field of DoC. Unfortunately, these scales are designed for awareness professionals and cannot meet the needs of family assessment.

Telephone follow-up is a purposeful interactive link between medical staff, patients, and their families using electronic information tools. Implementing a resource-saving follow-up form is easy to promote patients' recovery. Constructing a professional, simple, and feasible Telephone follow-up scale is the key to the effective follow-up implementation. Wannez et al. found in 282 patients with MCS in the behavior of the diagnosis, limiting CRS-R evaluation to the five most observed items (i.e., fixation, visual pursuit, reproducible response to command, automatic motor response, and localization to harmful stimuli) detected in 99% of MCS patients. If clinicians have only limited time to evaluate patients with DoC, we recommend that at least these five items of CRS-R be evaluated.

It has been reported that visual pursuit is very important in distinguishing MCS from VS patients, and the re-emergence of visual pursuit seems to be an early behavioral marker of patients' recovery from VS to MCS (10). This result was also confirmed in 2020. Martens et al. found that visual pursuit was the most common initial sign in their study of consciousness recovery in 79 patients with severe brain injury (18). In 2020, Carriere et al. conducted a multimodal analysis on patients with auditory localization, suggesting that auditory localization is a sign of MCS (17). Previous studies found that compared with VS, MCS patients had a robust perception of pain (32). The Nociception Coma Scale–Revised (NCS-R) was used to study the pain behavior for DoC patients (33), and it was found that patients' response to pain stimuli can effectively evaluate the state of consciousness for DoC patients (19).

Autonomic movement is a conscious behavior that we often observe. For example, DoC patient may scratch bedcovers, the nasal tube or catheter, and the body. These behaviors are triggered when the elementary sensory cortex detects an object touch or an external (e.g., object entering the visual field) stimulus. Neural signals are then sent downstream to the association cortex for further sensory encoding (e.g., what object is this) eventually reaching the motor cortex, which initiates specific motor sequences associated with the triggering stimulus (e.g., grasping the object).These processing steps indicate that awareness of self and environment must be preserved for such behaviors to be performed (18). Individual studies were confirmed in a study by Rem et al. who conducted an autonomous movement study in patients who had a stroke and found that patients who could cross their legs while sitting had significantly better post-injury recovery (34). Using these validated sensitive indicators as the main indicators of the telephone follow-up scale for DoC patients may increase the effectiveness of telephone follow-up.

Many recent studies have shown that the involvement of family members (35, 36) and home environment can enhance patients' awareness of behavioral performances. Due to the familiarity of patients' preference (37), patients' families can adopt more personalized stimulation. The deeper participation of family caregivers, the more observed subtle changes from patients. These cannot be replaced by medical staffs, so we should give full play to the role of patient's family members in the evaluation, to reduce the misdiagnosis rate of behavior evaluation.

In addition, communication by telephone also strengthens the link between patients and medical staff. It also has a guiding role in rehabilitating patients at home and in the community. A 2021 study on traumatic brain injury by Sebastiaan et al. found consistency in GOS-E test results for face-to-face, and telephone extensions (38). It indicated that telephone assessment was an effective alternative to face-to-face where this was not feasible. In addition, the telephone scale form is also convenient to use in the hospital environment. Using a telephone scale for follow-up can save time and timely understand the recovery status of patients.

A limitation of this study is that we included only behavioral indicators that have been proven repeatedly, while some promising new indicators that are being promoted, such as auditory localization (17); habituation of auditory startle reflex (39); spontaneous eye blinking (40); resistance to eye-opening; olfactory stimulation (20) still needs to be verified in larger samples and multi-center environments, so it is not included. In addition, being sensitive, simple, and easy to operate for family members is a very important goal of the telephone follow-up scale.

## Conclusion

Currently, most patients are in an environment with insufficient medical resources. Through telephone communication with patients' family members, patients' subtle changes can be timelier understood, and health guidance can be given to them. In this study, observing patients' family members and diagnosing professional evaluators will be used to enhance the objectivity of consciousness assessment.

## Ethics statement

The studies involving human participants were reviewed and approved by the Hangzhou Normal University (No. 2022044). The patients/participants provided their written informed consent to participate in this study.

## Author contributions

FS and JW conceived and designed the study. FS wrote the protocol and manuscript. WH and LC participated in the discussion of the study protocol. HD and SL substantially contributed to study supervision. All authors contributed to the article and approved the submitted version.
