# Supplementary material for: Study protocol: Developing telephone follow-up scale for patients with disorders of consciousness
Source: Front Public Health. 2023 Mar 30;11:1071008. doi: 10.3389/fpubh.2023.1071008 (PMC10097956; doi:10.3389/fpubh.2023.1071008)
Supplement: Supplementary file 1 [file Data_Sheet_1.pdf]

Appendix 1:

The "Telephone Follow-up Scale for Disorders of Consciousness patients" framework construction Expert Consultation Questionnaire

Dear Expert:

Hello! This study is to construct a "Telephone Follow-up Scale" for disorders of consciousness （DoC） patients to improve the rehabilitation data of prolonged DoC patients.

The lack of prognostic data on rehabilitation is the major factor affecting clinical treatment decisions and ethical issues in DoC patients. Consciousness assessment tools have been currently used in clinics. But such tools cannot meet the follow-up needs of prolonged DoC patients.

This telephone follow-up is a targeted link in medical staffs, patients, and their families through electronic information tools. It is an easy-to-implement and resource-saving follow-up form to promote patients' recovery in maximized scale, fast data-collecting and low cost. There are no related published papers in the field of DoC. **The purposes of this study are to construct a sensitive, professional, and simple telephone follow-up scale for DoC patients and to follow up the prognosis of long-term DoC patients, those who were transferred to community hospitals and at home, especially during their recovery of consciousness.**

To make the scale items more pertinent, in the early stage, the literature on behavior scale was retrieved and summarized, and the preliminary construction of the scale was completed. After literature research and expert argumentation, 2 first-level indicators, 9 second-level indicators and 31 third-level indicators were determined.

In view of your profound expertise and extensive clinical experience, we sincerely invite you to serve as the correspondence expert for this study. Please evaluate the importance, accuracy, and operability of each initial indicator according to the description, hope you can give us your valuable advice. To ensure the smooth progress of this research, please complete this questionnaire and return to **shoufangfang2022@163.com** before **July 01, 2022**. We will keep your inquiry results and information strictly confidential, and sincerely thank you for your support and help!

Contact with Fangfang Shou

at (+0086)-18268002931

International Unresponsive Wakefulness Syndrome and Consciousness Science Institute

Part 1: Expert Consultation Form for the "Telephone Follow-up Scale for DoC patients"

1. Questionnaire for expert consultation on evaluation indicators

Scoring instructions: Please rate each indicator according to the importance, accuracy, and operability, and write the corresponding score in the blank. If you think a certain indicator needs to be adjusted or revised, please write it in the column of "revision opinions"; If you have other opinions or add items, please add them in the column of "other opinions" and judge its importance, accuracy, and operability according to the same

principle.

1) Importance: Refers to the importance of a certain indicator in the evaluation of consciousness and functional recovery in DoC patients.

Scoring criteria: very important = 5, important = 4, general = 3, unimportant = 2, very unimportant = 1.

2) Accuracy: Refers to the appropriateness and accuracy of a certain indicator to reflect the content of consciousness and functional recovery of DoC patients.

Scoring criteria: strong accuracy = 3, general accuracy = 2, weak accuracy = 1.

3) Operability: Refers to the feasibility of a certain indicator in specific evaluation.

Scoring criteria: strong operability = 3, general operability = 2, weak operability = 1.

（1）First-level indicator score

**First-level indicator**	**Importance** （ very important = 5, important = 4, general = 3, unimportant = 2, very unimportant = 1.）	**Accuracy** （ strong accuracy = 3, general accuracy = 2, weak accuracy = 1.）	**Operability** （ strong operability = 3 , general operability = 2, weak operability = 1.）	**Revision opinions**
**1 Consciousness Recovery Dimension**<br>The patient's cognition of himself and the surrounding environment, the behavioral response to audio-visual, olfactory, tactile and pain, etc., and the recovery of the ability to communicate with others				

**2 Function Recovery Dimension**<br>Recovery of language and motor function, learning and completing certain adaptive tasks, self-care, and the ability to participate in recreational and occupational activities				
**Other opinions ：**				

（2）Second-level indicator score

**First-level indicator**	**Second-level indicator**	**Importance**<br>（ very important = 5, important = 4 , general = 3 , unimportant = 2 , very unimportant = 1.）	**Accuracy**<br>（ strong accuracy = 3 , general accuracy = 2 , weak accuracy = 1.）	**Operability**<br>（ strong operability = 3 , general operability = 2 , weak operability = 1.）	**Revision opinions**
**1Consciousness Recovery Dimension**	**1.1 Arousal level**<br>The patient has a sleep cycle, and different arousal levels are divided according to the stimuli before open the eyes				
	**1.2 Visual response**<br>Perform corresponding visual stimuli to judge the patient's response				

	**1.3 Auditory response**<br>Perform corresponding auditory stimuli to judge the patient's response				
	**1.4 Communication function**<br>Verbal (vocal) responses of patients with daily communication				
	**1.5 Motor function**<br>Muscles, joints and other related motor functions				
	**1.6 Tactile and pain response**<br>Perform corresponding tactile and pain stimuli to judge the patient's response				
	**1.7 Olfactory response**<br>Perform corresponding olfactory stimuli to judge the patient's response				
	**1.8 Emotional response**<br>Perform corresponding stimuli that the patient likes or dislikes to judge the patient's response				
**2Function Recovery Dimension**	**Prognostic functional recovery**<br>Recovery of self-care and the ability to participate in recreational and occupational activities				
**Other opinions ：**					

（3）Third-level indicator score

First-level indicator	Second-level indicator	Third-level indicator score	**Importance** （ very important = 5 , important = 4 , general = 3 , unimportant = 2 , very unimportant = 1.）	**Accuracy** （ strong accuracy = 3, general accuracy = 2, weak accuracy = 1.）	**Operability** （ strong operability = 3 , general operability = 2, weak operability = 1.）	Revision opinions
**1 Consciousness Recovery Dimension**	**1.1 Arousal level**	**1.1.1 Eye opening voluntarily**<br>Open eyes without external stimulation				
		**1.1.2 Eye opening with calling name**<br>Open eyes with gently calling for name or hearing a voice				
		**1.1.3 Eye opening with tactiling**<br>Open eyes with hard shake				
		**1.1.4. Eye opening with stinging**<br>Open eyes when stimulated by pain, and closed quickly when not stimulated				
		**1.1.5 No arousal**				

		Patients can't open eyes with any stimuli				
	**1.2 Visual response**	**1.2.1 Object Recognition**<br>Do the patient identify familiar people or objects?				
		**1.2.2 Visual Pursuit**<br>Do the patient's eyes follow people or objects?				
	**1.3 Auditory response**	**1.3.1 Movement to Command**<br>Can he follow commands and act accordingly? (Turning head, raising hand, blinking, and opening mouth, etc.)				
		**1.3.2 Localization to Sound**<br>Does patient turn his/her head or eyes to you when calling his/her name?				
		**1.3.3 Auditory Startle**<br>How does the patient respond to the startling sound (blinking, shaking, closing eyes, making sounds, etc.)				
	**1.4 Communication function**	**1.4.1 Normal communication**<br>Q&A may be slow (or slurred), but answers are correct				
		**1.4.2 Abnormal communication**<br>The patient can only vocalize and speak incomprehensible words				

		**1.4.3 Unable communication**<br>Patient can't make any sound				
	**1.5 Motor function**	**1.5.1 Functional object use**<br>Patient can use simple objects (cup, comb, pen, etc.)				
		**1.5.2 Automatic motor response**<br>Grabbing tubes (nasal feeding tubes, etc.), grasping sheets, scratching head, etc.				
		**1.5.3 Abnormal posture**<br>Whether the patient has rigid limbs and abnormal posture				
		**1.5.4 No response**<br>Unresponsive, muscle relaxation				
	**1.6 Tactile and pain response**	**1.6.1 Localization to pain**<br>Can the patient feel the pain points during massage or pain stimulation?				
		**1.6.2 pain escape**<br>Can the patient withdraw the flexion during massage or painful stimulation?				
		**1.6.3 No response**<br>Unresponsive to any stimulus				
	**1.7 Olfactory response**	**1.7.1 Olfactory Recognition**<br>Whether the patient smiles (frown) when they smell like (dislike) smells				
		**1.7.2 Olfactory Localization**<br>Will the patient turn his/her head to the smell				

		or move closer to the smell?				
	**1.8 Emotional response**	**1.8.1 Emotional expression**<br>Does the patient smile (frown) when they hear a sound that they like (dislike) or music?				
		**1.8.2 Familiar people recognition**<br>Does the patient shed tears or get emotional when seeing relatives?				
**2 Function Recovery Dimension**	**2.1 Prognostic functional recovery**	**2.1.1 Good recovery**<br>No residual disability associated with the conscious illness process				
		**3.1.2 Partial recovery**<br>With minor physical and/or intellectual disability				
		**2.1.3 Mild disability**<br>Disabled but available for some moderate work				
		**2.1.4 Moderate disability**<br>Disabled but available for some light work				
		**2.1.5 Severe disability**<br>Need help with some daily activities				
		**2.1.6 Very severe disability**<br>The patient cannot take care of himself and is completely dependent on others				
		**2.1.7 Vegetative state**				

		Patient is unconscious and the life entirely depends on others				
**Other opinions ：**						

Part 2: The questionnaire for basic information of experts

1、Expert authority

Please make a self-evaluation of the degree of authority (Cr) on your familiarity with the above indicators and the basis for scoring. Authority (Cr) includes judgment basis (Ca) and familiarity (Cs), Cr=(Ca + Cs)/2. Please tick "√" on the value that matches the actual situation

1) Your familiarity with the content of this consultation（Check 1 of the following 5）

**Very familiar**	**More familiar**	**Generally familiar**	**Unfamiliar**	**Very unfamiliar**
1	0.75	0.5	0.25	0

2) Your judgment on this evaluation indicator pool （The following 4 aspects need to be selected from "Large", "Medium" and "Small"）

**Judgment basis**	**The degree of influence on your judgment**		
	**Large**	**Medium**	**Small**
**（1）Theoretical analysis**	0.3	0.2	0.1
**（2）Practical experience**	0.5	0.4	0.3
**（3）Reference material**	0.1	0.1	0.1
**（4）Intuitive judgment**	0.1	0.1	0.1

2、Basic information of experts

1) Name：________________ 2) Work unit：_____________________

3) Gender：①Male ②Female 4) Age (years)：__________________________

5) Educational level：①Undergraduate ②Master ③PhD

6) Professional title：①Positive senior title ②Deputy senior title ③Intermediate title ④Junior professional title and below

7) Your current major professional field and working years（If more than one field, please indicate the age of the two professional fields）

（1）professional field 1：_____________； Years：____________； （2）professional field 2：_____________； Years：____________；

Sincerely thank you for your support and help,

Wish you good health and good work!
